# Predictive value of individual Sequential Organ Failure Assessment sub-scores for mortality in the cardiac intensive care unit

**DOI:** 10.1371/journal.pone.0216177

**Published:** 2019-05-20

**Authors:** Jacob C. Jentzer, Courtney Bennett, Brandon M. Wiley, Dennis H. Murphree, Mark T. Keegan, Gregory W. Barsness

**Affiliations:** 1 Department of Cardiovascular Medicine, Mayo Clinic, Rochester, Minnesota, United States of America; 2 Division of Pulmonary and Critical Care Medicine, Department of Internal Medicine, Mayo Clinic, Rochester, Minnesota, United States of America; 3 Department of Health Sciences Research, Mayo Clinic, Rochester, Minnesota, United States of America; 4 Department of Anesthesiology and Perioperative Medicine, Mayo Clinic, Rochester, Minnesota, United States of America; Wayne State University, UNITED STATES

## Abstract

**Purpose:**

To determine the impact of Sequential Organ Failure Assessment (SOFA) organ sub-scores for hospital mortality risk stratification in a contemporary cardiac intensive care unit (CICU) population.

**Materials and methods:**

Adult CICU admissions between January 1, 2007 and December 31, 2015 were reviewed. The SOFA score and organ sub-scores were calculated on CICU day 1; patients with missing SOFA sub-score data were excluded. Discrimination for hospital mortality was assessed using area under the receiver-operator characteristic curve (AUROC) values, followed by multivariable logistic regression.

**Results:**

We included 1214 patients with complete SOFA sub-score data. The mean age was 67 ± 16 years (38% female); all-cause hospital mortality was 26%. Day 1 SOFA score predicted hospital mortality with an AUROC of 0.72. Each SOFA organ sub-score predicted hospital mortality (all p <0.01), with AUROC values of 0.53 to 0.67. On multivariable analysis, only the cardiovascular, central nervous system, renal and respiratory SOFA sub-scores were associated with hospital mortality (all p <0.01). A simplified SOFA score containing the cardiovascular, central nervous system and renal sub-scores had an AUROC of 0.72.

**Conclusions:**

In CICU patients with complete SOFA sub-score data, risk stratification for hospital mortality is determined primarily by the cardiovascular, central nervous system, renal and respiratory SOFA sub-scores.

## Introduction

Risk prediction scores have guided care in the cardiac intensive care unit (CICU) since Killip, et al. reported their classification of patients with acute myocardial infarction.[1] The CICU population has evolved to include patients with acute and chronic multi-organ dysfunction and superimposed cardiac pathology, similar to other intensive care unit (ICU) populations.[2–7] Risk stratification models allow prediction of adverse outcomes in this increasingly complex CICU patient population in order to facilitate care planning and therapeutic intervention.[3, 4, 8] The use of disease-specific risk prediction scores in the CICU is limited by the presence of undifferentiated clinical syndromes in patients with multiple acute and chronic cardiovascular disease processes, making general ICU severity of illness scoring models potentially advantageous.[2, 6, 8–11]

The Sequential Organ Failure Assessment (SOFA) score is an illness severity score developed in patients with sepsis, including a 4-point assessment of dysfunction in each of 6 organ systems (central nervous system, cardiovascular, respiratory, renal, liver and coagulation).[11–13] The SOFA score contains fewer variables and is simpler to calculate compared to other ICU risk prediction models, yet it can accurately predict short-term mortality in CICU populations.[13–15] We previously reported very good discrimination for hospital mortality using the SOFA score on the first CICU day in our CICU population, although calibration was suboptimal.[15] The cardiovascular and renal SOFA organ sub-scores had the highest discrimination for short-term mortality in our prior study. However, data to calculate the respiratory and liver SOFA sub-scores were available in fewer than one-third of patients; as is customary in such models, missing data were imputed as normal.[15] The absence of available data for calculating ICU severity of illness scoring models influences model performance by underestimating illness severity and mortality risk, raising questions about the accuracy of the SOFA score in patients with missing data.[16]

The purpose of this study was to determine the relative contribution of each individual SOFA organ sub-score for prediction of mortality in CICU patients without any missing SOFA sub-score data, in order to facilitate potential future modification of the SOFA score to better fit the CICU population. Additionally, we sought to further explore the importance of missing data for mortality risk prediction using the SOFA score in CICU patients, as highlighted in our prior work.

## Materials and methods

This study was approved by the Mayo Clinic Institutional Review Board under an exception from informed consent as posing minimal risk to patients. This is a subset analysis of a historical cohort analysis utilizing an institutional database of patients admitted to the CICU at the Mayo Clinic Hospital, St. Mary’s Campus, as previously described.[15] The Mayo Clinic CICU is a closed 16-bed unit serving critically-ill cardiac medical patients, not including postoperative cardiac surgery patients or patients receiving extracorporeal membrane oxygenation support. Consultation by a Critical Care Medicine physician is provided for assistance in management of patients with respiratory failure. Unique adult patients ≥ 18 years old admitted to the CICU between January 1, 2007 and December 31, 2015 were identified and data from the first CICU admission were used.[17] Patients admitted to the CICU prior to January 1, 2007, patients still hospitalized on December 31, 2015 and patients who did not provide Minnesota Research Authorization under Minnesota state law were excluded from the initial study population. We excluded patients in whom any of the individual SOFA organ sub-scores could not be calculated due to the presence of missing data points; for SOFA organ sub-scores (such as cardiovascular and renal) based on multiple data points, patients missing either of the required data points were excluded.

As described previously, demographic and laboratory data and use of invasive ventilation and catecholamine infusions during the first 24 hours of CICU admission were collected.[15] The SOFA score (with individual SOFA organ sub-scores), Acute Physiology and Chronic Health Evaluation (APACHE)-III score and Oxford Acute Severity of Illness Score (OASIS) were generated using data in the electronic medical record system from the first 24 hours of CICU admission; for the APACHE-III score and OASIS, missing variables were imputed as normal (score of 0) as the default.[18–21] Total SOFA scores were automatically calculated on each day a patient remained in the CICU, and the mean and maximum of all SOFA scores up to the first week in the CICU were recorded. The Charlson Comorbidity Index (CCI) was calculated electronically.[22] Sepsis was identified using a previously-validated electronic algorithm.[23] Relevant cardiovascular hospital discharge diagnoses were determined using ICD-9 diagnostic codes.

The primary study endpoint was all-cause hospital mortality; secondary endpoints included all-cause CICU mortality and 30-day mortality. Mortality data were extracted from Mayo Clinic electronic databases, the state of Minnesota electronic death certificates and the Rochester Epidemiology Project database, as previously described.[24] Categorical variables are reported as number (%), and the chi-squared test was used to compare groups. Continuous variables are reported as mean (± standard deviation, SD), and Student’s t-test was used to compare groups. Univariate analysis was performed using continuous variables as predictors of mortality, and the area under the receiver operating characteristic curve (AUROC) values were determined. AUROC confidence intervals (CI) were calculated via 2000 bootstrap samples, and AUROC values were compared between scores using the DeLong test. A logistic regression model was created for each score to determine calibration for hospital mortality using the Hosmer-Lemeshow statistic. Multivariate analysis was performed using logistic regression including each individual SOFA sub-score as a continuous variable. Two-tailed P values <0.05 were considered statistically significant. Statistical analyses were performed using JMP version 13.0 Pro (SAS Institute, Cary, NC) and R version 3.4.2 (https://www.r-project.org/).

## Results

We screened 12904 adult admissions to the CICU during the study period and excluded 2900 patients, yielding an initial population of 10004 patients (S1 Fig).[15] Day 1 SOFA score data were available in 9989 (99.9%) patients, including the cardiovascular sub-score in 9971 (99.7%), central nervous system sub-score in 9674 (96.7%), renal sub-score in 9431 (94.3%), coagulation sub-score in 9300 (93.0%), respiratory sub-score in 3128 (31.3%) and liver sub-score in 2651 (26.5%). The majority of patients had available data for 4 (n = 4985, 49.8%) or 5 (n = 3054, 30.5%) SOFA sub-scores; 751 (7.5%) patients had available data for 3 or fewer SOFA sub-scores. The 1214 (12.1%) patients who had data available for all 6 SOFA sub-scores comprised the final study population (S1 Fig). The remaining 8790 (87.9%) patients with missing data for at least 1 SOFA sub-score were excluded.

In the final study population, the mean age was 66.7±15.0 years and 459 (37.8%) patients were female (Table 1). The final study population differed significantly from excluded patients with missing SOFA sub-score data, with higher illness severity and different cardiovascular discharge diagnoses (Table 1). The mean SOFA score in the final study population was 8.1±3.6 compared to 2.9±3.6 in the excluded patients with missing SOFA sub-score data (p <0.001), and the SOFA score distribution was shifted towards higher SOFA scores in the final study population (S2 Fig). The mean SOFA score of 8 in the final study population corresponds to the 90^th^ percentile for the initial population.[15] In the final study population, all-cause CICU mortality occurred in 208 (17.1%) patients, hospital mortality occurred in 311 (25.6%) patients, and 30-day mortality occurred in 356 (29.3%) patients; these mortality rates are higher than previously reported for the initial study population.[15] Short-term mortality was significantly (p <0.001) higher in the final study population compared to excluded patients with missing SOFA sub-score data (S3 Fig), as were CICU and hospital length of stay (Table 1). Notably, there was a U-shaped relationship between number of SOFA sub-scores with available data and short-term mortality (S4 Fig).

**Table 1:** Baseline characteristics of final study population compared to eligible patients excluded due to incomplete SOFA sub-score data.

	Final study population with complete SOFA sub-score data N = 1214 (12.1%)		Patients excluded due to incomplete SOFA sub-score data N = 8790 (87.9%)		
	#	N(%) or Mean±SD	#	N(%) or Mean±SD	P value
***Demographics***					
**Age**	1214	66.7±15.0	8790	67.5±15.2	0.0794
**Female**	1214	459 (37.8%)	8790	3287 (37.4%)	0.7799
**White race**	1214	1097 (90.4%)	8790	8139 (92.6%)	0.0062
**BMI (kg/m**^**2**^**)**	1200	30.3±7.8	8684	29.4±7.0	0.0001
***Severity of illness scores***					
**APACHE-III score**	1214	87.57±30.67	8790	57.34±22.13	<0.0001
**OASIS score**	1214	36.19±11.58	8790	23.83±9.21	<0.0001
**Total SOFA score**	1214	8.14±3.63	8775	2.93±2.60	<0.0001
**Respiratory SOFA score**	1214	2.68±1.00	1914	2.43±1.08	<0.0001
**Coagulation SOFA score**	1214	0.54±0.82	8086	0.30±0.60	<0.0001
**Liver SOFA score**	1214	0.43±0.79	1437	0.30±0.69	<0.0001
**Cardiovascular SOFA score**	1214	2.04±1.28	8757	1.13±0.75	<0.0001
**CNS SOFA score**	1214	1.14±1.56	8460	0.23±0.75	<0.0001
**Renal SOFA score**	1214	1.31±1.25	8217	0.76±1.08	<0.0001
***Procedures and therapies***					
**Mechanical ventilator day 1**	1214	611 (50.3%)	8790	787 (9.0%)	<0.0001
**Catecholamines day 1**	1214	604 (49.8%)	8775	1166 (13.3%)	<0.0001
**# catecholamines day 1**	1214	0.80±0.98	8775	0.18±0.50	<0.0001
**New dialysis start**	1214	79 (6.5%)	8790	244 (2.8%)	<0.0001
**Inpatient coronary angiogram**	1214	563 (46.4%)	8790	4721 (53.7%)	<0.0001
**Inpatient PCI**	1214	284 (23.4%)	8790	3143 (35.8%)	<0.0001
**Intra-aortic balloon pump**	1214	219 (18.0%)	8790	646 (7.4%)	<0.0001
**Pulmonary artery catheter**	1214	199 (16.4%)	8790	522 (5.9%)	<0.0001
**Transfusion in CICU**	1214	306 (25.2%)	8790	867 (9.9%)	<0.0001
***Comorbidities***					
**Prior dialysis**	1214	109 (9.0%)	8790	462 (5.3%)	<0.0001
**Charlson comorbidity index**	1212	2.79±2.82	8766	2.32±2.58	<0.0001
**History of myocardial infarction**	1212	248 (20.5%)	8766	1732 (19.8%)	0.5647
**History of heart failure**	1212	299 (24.7%)	8766	1654 (18.9%)	<0.0001
**History of stroke**	1212	176 (14.5%)	8766	1053 (12.0%)	0.0127
**History of chronic kidney disease**	1212	302 (24.9%)	8766	1729 (19.7%)	<0.0001
**History of diabetes mellitus**	1212	419 (34.6%)	8766	2418 (27.6%)	<0.0001
**History of cancer**	1212	254 (21.0%)	8766	1881 (21.5%)	0.6903
**History of lung disease**	1212	274 (22.6%)	8766	1670 (19.0%)	0.0034
***Discharge diagnoses***					
**Cardiogenic shock**	1214	299 (24.6%)	8780	540 (6.2%)	<0.0001
**Cardiomyopathy**	1214	215 (17.7%)	8780	1153 (13.1%)	<0.0001
**Heart failure**	1214	551 (45.4%)	8780	5587 (63.6%)	<0.0001
**Atrial fibrillation**	1214	481 (39.6%)	8780	2674 (30.5%)	<0.0001
**Cardiac arrest**	1214	282 (23.2%)	8780	525 (6.0%)	<0.0001
**Acute coronary syndrome**	1214	451 (37.2%)	8780	3820 (43.5%)	<0.0001
**Coronary artery disease**	1214	683 (56.3%)	8780	5381 (61.3%)	0.0009
**Sepsis**	1214	541 (44.6%)	8790	1126 (12.8%)	<0.0001
***Admission vital signs***					
**Admission systolic BP (mmHg)**	1211	119.3±28.7	8728	123.6±25.9	<0.0001
**Admission diastolic BP (mmHg)**	1161	68.0±19.6	8462	69.7±16.6	0.0051
**Admission mean BP (mmHg)**	1161	82.0±20.9	8462	83.7±17.6	0.0067
**Admission heart rate (per min)**	1211	90.9±24.6	8732	80.9±22.9	<0.0001
**Admission respiratory rate (per min)**	1160	20.7±6.8	8436	18.1±5.5	<0.0001
**Admission SpO**_**2**_ **(%)**	1211	93.5±9.4	8728	96.0±5.3	<0.0001
**Admission GCS**	1209	10.4±5.0	8523	14.3±2.5	<0.0001
***Length of stay***					
**Inpatient days prior to CICU**	1214	0.59±2.26	8790	0.70±2.65	0.1097
**CICU LOS**	1214	3.84±3.77	8790	2.29±4.63	<0.0001
**Hospital LOS**	1214	11.01±12.56	8790	7.47±12.17	<0.0001

Data displayed as N (%) or mean ± standard deviation, with P value for between-groups comparison using chi squared or Student *t* test.

Abbreviations: APACHE, Acute Physiology and Chronic Health Evaluation; BMI, body mass index; BP, blood pressure; CICU, cardiac intensive care unit; CNS, central nervous system; GCS, Glasgow coma scale; LOS, length of stay; OASIS, Oxford Acute Severity of Illness Score; PCI, percutaneous coronary intervention; SOFA, Sequential Organ Failure Assessment; SpO_2_, pulse oximetry.

**Fig 1:**
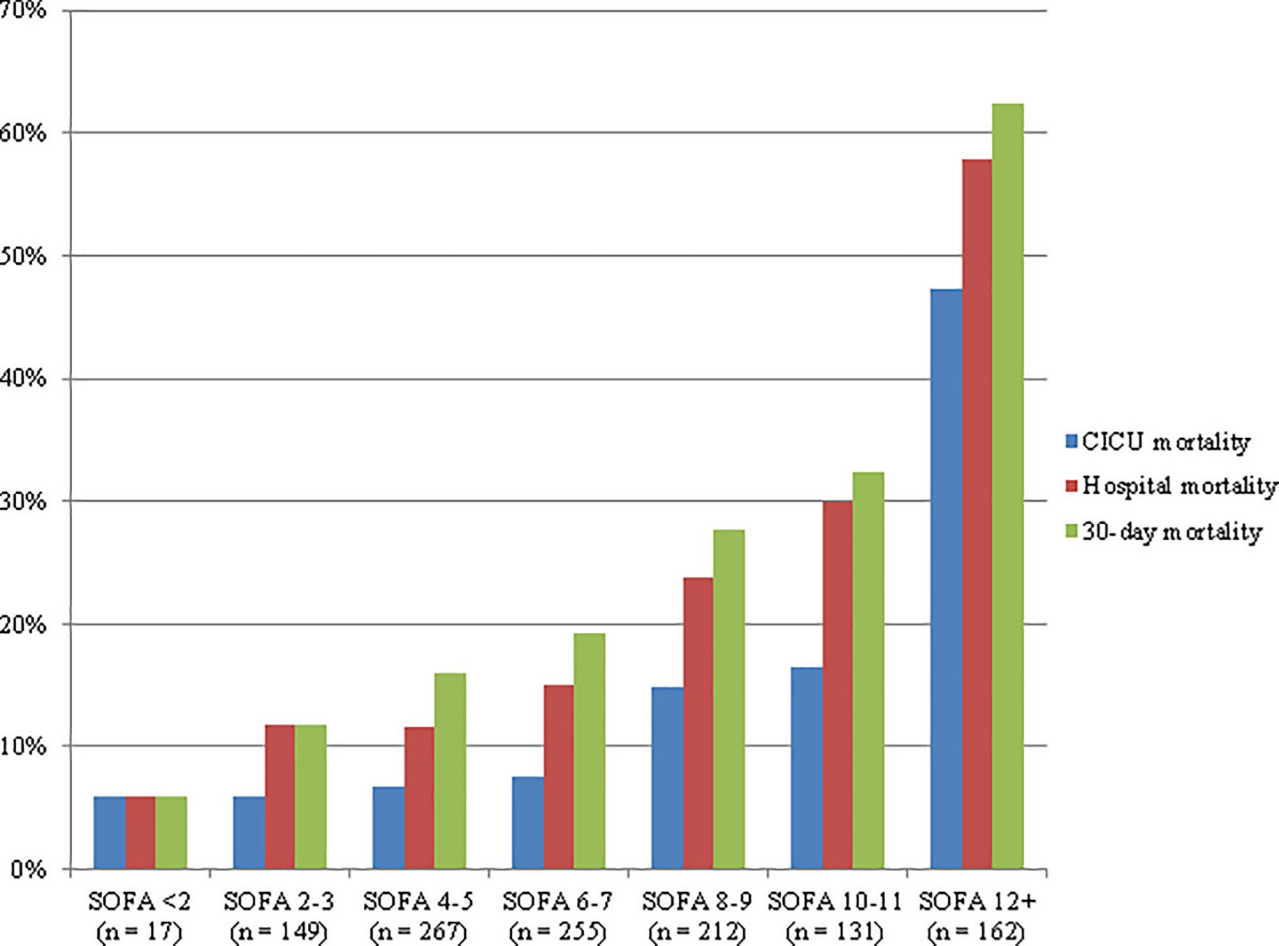
Short-term mortality as a function of Day 1 SOFA score in the final study population. P <0.001 for trend.

Short-term mortality increased progressively with rising Day 1 SOFA score in the final study population (Fig 1). The Day 1 SOFA score was a univariate predictor of hospital mortality in the final study population (OR 1.28, 95% CI 1.23–1.34, AUROC 0.72, p <0.001; Table 2). As shown in Table 2, the discriminative capacity of the APACHE-III score for hospital mortality (AUROC 0.79; p <0.001 by DeLong test) was higher than the Day 1 SOFA score in the final study population; the OASIS score performed similarly to the Day 1 SOFA score (AUROC 0.73; p >0.05 by DeLong test). In the 1,098 (90.4%) patients without missing data for calculating OASIS (i.e. in whom imputation of missing data was not necessary), the AUROC was 0.77 (p = 0.01 by DeLong test compared with SOFA). Calibration of the APACHE-III score using the Hosmer-Lemeshow statistic (Table 2) was good (p = 0.157), while calibration of the SOFA score was poor (p = 0.037); calibration of OASIS was borderline (p = 0.055). The mean and maximum SOFA score during the first 2 CICU days outperformed the Day 1 SOFA for prediction of hospital mortality (p <0.05 by DeLong test). The mean SOFA score during the first week in the CICU had the highest AUROC value of any of the scores tested (0.82) and had good calibration (p = 0.253), but the AUROC was not significantly different than APACHE-III (p = 0.07 by DeLong test). Among the 989 (81.5%) patients remaining in the CICU for >1 day, the 131 patients (13.2%) with a rising Day 2 SOFA had increased hospital mortality (36.6% vs. 19.8%, OR 2.34, 95% CI 1.58–1.37, p <0.001).

**Table 2:** Univariate analysis of illness severity scores as predictors of hospital mortality in the final study population (n = 1214).

Risk score	Unit OR	OR 95% CI	AUROC	AUROC 95% CI	P value*
**APACHE-III score**	1.039	1.034–1.045	0.788	0.760–0.817	0.157
**OASIS score**	1.082	1.068–1.097	0.728	0.695–0.761	0.055
**Day 1 SOFA score**	1.284	1.233–1.337	0.725	0.691–0.759	0.037
**Maximum Day 1+2 SOFA score**	1.334	1.278–1.394	0.753	0.721–0.785	0.009
**Mean Day 1+2 SOFA score**	1.393	1.329–1.460	0.777	0.747–0.808	0.737
**Maximum week 1 SOFA score**	1.367	1.308–1.430	0.778	0.747–0.808	0.033
**Mean week 1 SOFA score**	1.490	1.414–1.571	0.815	0.787–0.844	0.253
**Respiratory SOFA sub-score**	1.636	1.402–1.909	0.625	0.593–0.658	0.883
**Coagulation SOFA sub-score**	1.221	1.050–1.420	0.531	0.498–0.564	0.989
**Liver SOFA sub-score**	1.277	1.095–1.490	0.535	0.505–0.566	0.975
**Cardiovascular SOFA sub-score**	1.649	1.486–1.830	0.669	0.635–0.703	0.745
**CNS SOFA sub-score**	1.356	1.253–1.469	0.615	0.580–0.650	0.129
**Renal SOFA sub-score**	1.632	1.472–1.809	0.674	0.641–0.708	0.180
**SOFA without Respiratory sub-score**	1.306	1.249–1.365	0.717	0.683–0.750	0.094
**SOFA without Coagulation sub-score**	1.317	1.261–1.377	0.729	0.695–0.763	0.329
**SOFA without Liver sub-score**	1.300	1.245–1.356	0.727	0.693–0.761	0.323
**SOFA without Cardiovascular sub-score**	1.328	1.263–1.395	0.708	0.674–0.743	0.751
**SOFA without CNS sub-score**	1.336	1.271–1.405	0.716	0.682–0.751	0.620
**SOFA without Renal sub-score**	1.272	1.217–1.330	0.693	0.657–0.729	0.019
**Simplified SOFA score (3 sub-scores)^**	1.372	1.304–1.444	0.723	0.690–0.757	0.201
**Simplified SOFA score (4 sub-scores)#**	1.323	1.265–1.384	0.728	0.695–0.762	0.502

All scores are from CICU day 1 unless otherwise specified.

* P value is for Hosmer-Lemeshow statistic; p values < 0.05 reflect poor calibration.

^ Cardiovascular, central nervous system, renal

# Cardiovascular, central nervous system, renal, respiratory

Abbreviations: APACHE, Acute Physiology and Chronic Health Evaluation; AUROC, area under the receiver-operator characteristic curve; CNS, central nervous system; OASIS, Oxford Acute Severity of Illness Score; SOFA, Sequential Organ Failure Assessment.

**Fig 2:**
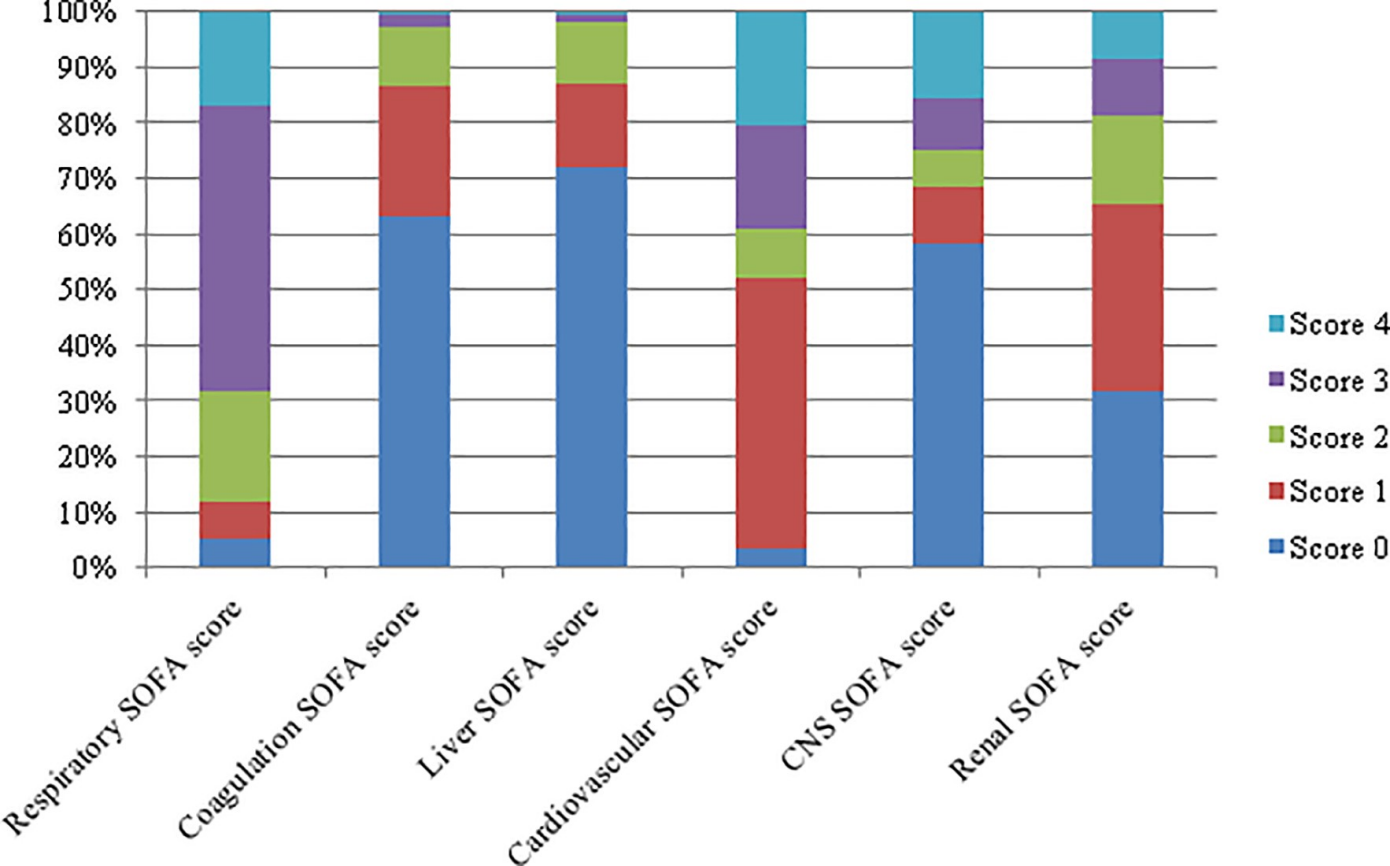
Distribution of individual SOFA organ sub-scores in final study population.

The distribution of individual SOFA sub-scores in the final study population is shown in Fig 2. Each of the individual organ sub-scores was a univariate predictor of mortality (Table 2; all p <0.01). The cardiovascular and renal sub-scores had the highest AUROC values (0.67) for hospital mortality and the coagulation sub-score had the lowest AUROC value (0.53). Limited SOFA scores were calculated by omitting each of the SOFA organ sub-scores individually and discrimination for hospital mortality was assessed. Removal of the coagulation or liver sub-scores had minimal impact on the AUROC values for hospital mortality, while removal of the other sub-scores had a greater impact (Table 2). The lowest AUROC value occurred when the renal sub-score was removed from the Day 1 SOFA score. In an exploratory analysis examining these modified SOFA scores among patients excluded from the study due to missing data, AUROC values for all tested scores were similar or higher compared to AUROC values seen in patients included in the final study population (S1 Table).

On multivariate analysis including all 6 SOFA organ sub-scores as predictors of hospital mortality, only the cardiovascular, central nervous system, respiratory and renal sub-scores were significant predictors of hospital mortality (Table 3). The AUROC value of 0.74 for hospital mortality in the regression model was essentially unchanged when the coagulation and/or liver sub-scores were removed. When the coagulation sub-score was removed from the regression model, the liver sub-score became marginally significant as a predictor of hospital mortality (p = 0.048).

**Table 3:** Multivariate analysis of Day1 SOFA sub-scores as predictors of hospital mortality in primary study population using logistic regression. AUROC of the multivariate model was 0.738 for hospital mortality.

Variable	Unit OR	95% CI	Estimate	SE	Chi-squared	P value
**Respiratory SOFA sub-score**	1.306	1.102–1.547	0.267	0.086	9.53	0.0020
**Coagulation SOFA sub-score**	1.007	0.844–1.202	0.007	0.090	0.01	0.9342
**Liver SOFA sub-score**	1.189	0.991–1.427	0.173	0.093	3.47	0.0625
**Cardiovascular SOFA sub-score**	1.347	1.199–1.513	0.298	0.059	25.13	<0.0001
**CNS SOFA sub-score**	1.217	1.112–1.331	0.196	0.046	18.21	<0.0001
**Renal SOFA sub-score**	1.519	1.360–1.697	0.418	0.056	54.71	<0.0001

Abbreviations: AUROC, area under the receiver-operator characteristic curve; CNS, central nervous system; SOFA, Sequential Organ Failure Assessment.

A simplified SOFA score including only the 4 SOFA organ sub-scores (cardiovascular, central nervous system, renal and respiratory) that were significantly predictive of hospital mortality on multivariate analysis had similar discriminative capacity compared to the Day 1 SOFA score for hospital mortality in the final study population (AUROC 0.73; Table 2); the AUROC value of this simplified SOFA score for the initial population was 0.83. Inclusion of only the cardiovascular, central nervous system and renal sub-scores again performed similarly in the final population (AUROC 0.72; Table 2); the AUROC value for the initial population was 0.81. Calibration of both of these simplified SOFA scores was good (p >0.05).

## Discussion

This is the first study to explore the predictive value of individual SOFA organ sub-scores for short-term mortality in a contemporary CICU population with complete data availability. These CICU patients with available data to calculate all 6 SOFA organ sub-scores constituted a cohort of severely ill patients with hospital mortality exceeding 25%. Among these high-risk patients, the Day 1 SOFA score had good discrimination for hospital mortality, although discrimination was lower than previously reported, and calibration was poor.[15] Removal of the cardiovascular and renal SOFA sub-scores had the greatest effect on discrimination of hospital mortality. Only the cardiovascular, central nervous system, respiratory and renal sub-scores were independently predictive of hospital mortality on multivariate analysis. Removing the coagulation and liver SOFA sub-scores did not substantially impact discrimination. Simplified SOFA scores including the cardiovascular, central nervous system and renal sub-scores (with or without the respiratory sub-score) had similar discrimination for hospital mortality as the original SOFA score in this selected cohort.

These findings expand on our prior study demonstrating that the Day 1 SOFA score had very good discrimination (AUROC value of 0.83) for hospital mortality in unselected CICU patients.[15] The availability of complete data for calculating the SOFA score significantly impacted its discrimination for hospital mortality, with paradoxically lower discrimination in patients with available data for all 6 SOFA organ sub-scores compared to the initial population or patients excluded due to missing data.[15] Patients in this study with complete SOFA sub-score data had higher illness severity, leading to lower discrimination for mortality by the SOFA score and other risk scores. Because ICU risk scores differentiate high-risk from low-risk patients, model performance would be expected to decrease in a sicker population. A prior study by Afessa, et al. demonstrated that individual variables used to calculate the Acute Physiology Score component of the APACHE score were more likely to be missing in less-sick patients, and patients without any missing data had the highest short-term mortality.[16]

We report a novel, U-shaped association between the number of SOFA sub-scores with available data and short-term mortality, with higher mortality in the small number of patients missing data for 3 or more SOFA sub-scores as well as among patients who had available data for the respiratory and liver SOFA sub-scores. This association between laboratory testing patterns and mortality mirrors the prior study by Afessa, et al. reporting that patients with a measured serum bilirubin or albumin level (fewer than 20% of all patients) had higher observed mortality.[16] In the study by Afessa, et al. the number of missing variables was associated with increased mortality after correcting for the APACHE-III score using multivariate analysis, yet the observed-to-expected mortality appeared to be lower in patients with complete data.[16]

Prior studies comparing the SOFA score with the APACHE score in general CICU populations have demonstrated similar discrimination for mortality.[14, 15] Our prior study showed very good discrimination and poor calibration by both the SOFA and APACHE-III scores, but the APACHE-III score performed better in the high-risk subgroup included in the present study.[15] Paradoxically, while the discrimination as measured by the AUROC value of the SOFA score was lower in the population without missing data, the discrimination AUROC value of OASIS was higher in patients without missing data. This divergent effect of missing data on the performance of SOFA and OASIS is novel, whereas missing data has been previously shown to decrease discrimination by the APACHE-III score.[16] Notably, in our prior study, the AUROC for most SOFA sub-scores likewise decreased when missing data were imputed as normal.[15] Therefore, we hypothesize that the lower discrimination by the Day 1 SOFA score in this cohort compared to our prior study cohort may be due to higher observed mortality in this cohort, especially among patients with low SOFA scores.[15] Unlike the SOFA score, the APACHE-III score retained very good discrimination for hospital mortality in this study population compared to our prior study (AUROC 0.79 vs. 0.82).[15] Superior risk prediction by the APACHE-III score in the selected subgroup represented in this study may reflect the greater number of variables in the APACHE-III model, potentially allowing refinement of risk prediction beyond the simpler SOFA score; improved prediction by models containing greater numbers of variables has previously been demonstrated.[10, 19] The major advantages of the SOFA score include its simplicity and ease of calculation, which allows daily SOFA score calculations to trend illness severity over time.[10, 15] However, these advantages are only relevant insofar as risk prediction remains robust; we hypothesize that the SOFA score may be more useful for distinguishing high-risk from low-risk patients, rather than further stratifying the high-risk patients.

Not all of the individual SOFA organ sub-scores are equally relevant for mortality risk prediction in CICU patients.[15] Because the original intent of the SOFA score was to prognosticate in patients with sepsis, the organ failure variables included in the SOFA score are reflective of those commonly seen in sepsis and may be less relevant in CICU patients.[10–13] Notably, there was a 45% prevalence of sepsis in this cohort of CICU patients with complete SOFA organ sub-score data. The coagulation and liver SOFA sub-scores were not independently associated with hospital mortality and therefore contributed little to risk prediction in this population, which is not surprising given the low prevalence of significant thrombocytopenia and hyperbilirubinemia. In this cohort, the respiratory (36%), cardiovascular (26%) and renal (15%) sub-scores contributed the most to the total SOFA score, while the coagulation and liver sub-scores together contributed only 11% to the total SOFA score.

The impact of individual SOFA organ sub-scores on overall mortality prediction has not been previously explored in CICU patients, apart from our prior study.[15] Knox, et al. demonstrated that the central nervous system SOFA sub-score (i.e. GCS) dominated the predictive value of the SOFA score in a mixed ICU population.[25] Toma, et al. used computer modeling to determine that the central nervous system sub-score was the most important predictor of mortality in general ICU patients, followed by the cardiovascular and renal sub-scores.[26] In our prior study, the cardiovascular and renal sub-scores had the highest AUROC and OR values for hospital mortality, followed by the central nervous system sub-score; when missing data were imputed as normal, the respiratory sub-score had the highest AUROC value, followed by the cardiovascular sub-score.[15]

This retrospective single-center cohort study has a number of limitations, including the possibility of unmeasured confounders and potential bias due to local practice patterns. Our patient population may be distinct from other centers, as reflected by a lower hospital death rate and rate of acute coronary syndromes in the initial population than most prior CICU studies.[2, 5, 6, 14] This study included the minority of highly-selected patients who had available data for calculating each of the SOFA sub-scores, with evidence of selection bias whereby these sicker patients were more likely to have serum bilirubin and/or arterial blood gases measured. Because patients with complete SOFA sub-score data available differed substantially from other CICU patients, conclusions about the performance of the SOFA score and relative predictive value of individual SOFA sub-scores in this selected subgroup may not be broadly applicable. Our analysis was limited by data missingness, which was not random but instead associated with illness severity. While we could have performed multiple imputation to account for this missing data, imputation of missing data as normal is the recommended and accepted methodology for dealing with missing data in prognostic scoring systems. This approach provides a more parsimonious estimate of model performance, but can be associated with inaccuracy of the prognostic models at the extremes of illness severity.[10, 16] Due to the potential non-randomness of missing data, a complete-case subgroup analysis, as performed in this study, has important limitations when compared to multiple imputation of missing data within the entire population. In addition, we did not have individual SOFA sub-scores available for subsequent CICU days to assess prediction at later time points; notably, the use of mean or maximum SOFA scores during the first week did not outperform the APACHE-III score.

In conclusion, CICU patients with the availability of complete data to calculate all 6 SOFA organ sub-scores are a high-risk population, reflecting a bias toward more laboratory testing in sicker patients. Discrimination of the SOFA and OASIS scores for hospital mortality was lower in this cohort than reported in the initial population; the APACHE-III score had the best performance of the scores we examined. The renal and cardiovascular SOFA sub-scores contribute the most to mortality prediction in these CICU patients, and the liver and coagulation SOFA sub-scores contribute little to mortality prediction. These findings emphasize the impact of data availability on performance of the SOFA score, and highlight the potential to refine the SOFA score for mortality risk prediction in CICU patients by replacing the current coagulation and liver SOFA sub-scores with variables more predictive of mortality in CICU populations. We suggest that future prospective studies using the SOFA score as a marker of illness severity use standardized methods for ensuring complete data availability to calculate each of the organ sub-scores, to avoid the limitations of missing data described herein. The suboptimal performance of the SOFA score in this study emphasizes the limitations of using the SOFA score in CICU populations and the need to develop better risk prediction models for CICU patients. Future studies are needed to determine the real-world performance of the SOFA score in CICU patients. This work may ultimately facilitate the future development of a CICU-specific SOFA-derived score or novel CICU-specific risk score that will be more widely applicable to CICU populations.

## Supporting information

S1 Fig Flow diagram demonstrating reasons for patient exclusion from the final study population. (TIF)

S2 Fig Distribution of SOFA scores in final study population and patients excluded due to incomplete SOFA sub-score data. P<0.001 between groups.
(TIF)

S3 Fig Short-term mortality in the final study population and patients excluded due to incomplete SOFA sub-score data. P <0.001 between groups.
(TIF)

S4 Fig Short-term mortality as a function of number of individual SOFA sub-scores with available data. P<0.001 between groups.
(TIF)

S1 Table Univariate analysis of Day 1 illness severity scores as predictors of hospital mortality in patients excluded from the final study population due to missing SOFA sub-score data (n = 8790). For this analysis, all missing data were imputed as normal. All p values <0.001.
(DOCX)

S1 Data Minimal data set supplemental excel spreadsheet file. (XLSX)

## Abbreviations

APACHE: Acute Physiology and Chronic Health Evaluation
AUROC: area under the receiver-operator characteristic curve
BMI: body mass index
CCI: Charlson Comorbidity Index
CI: confidence interval
CICU: cardiac intensive care unit
CKD: chronic kidney disease
ICU: intensive care unit
LOS: length of stay
OASIS: Oxford Acute Severity of Illness Score
OR: odds ratio
SOFA: Sequential Organ Failure Assessment

## References

[1] Killip T 3rd, Kimball JT: Treatment of myocardial infarction in a coronary care unit. A two year experience with 250 patients. Am J Cardiol 1967, 20(4):457–464. 6059183 10.1016/0002-9149(67)90023-9

[2] Katz JN, Shah BR, Volz EM, Horton JR, Shaw LK, Newby LK, et al: Evolution of the coronary care unit: clinical characteristics and temporal trends in healthcare delivery and outcomes. Crit Care Med 2010, 38(2):375–381. 10.1097/CCM.0b013e3181cb0a63 20029344

[3] Katz JN, Minder M, Olenchock B, Price S, Goldfarb M, Washam JB, et al: The Genesis, Maturation, and Future of Critical Care Cardiology. J Am Coll Cardiol 2016, 68(1):67–79. 10.1016/j.jacc.2016.04.036 27364053

[4] Morrow DA, Fang JC, Fintel DJ, Granger CB, Katz JN, Kushner FG, et al: Evolution of critical care cardiology: transformation of the cardiovascular intensive care unit and the emerging need for new medical staffing and training models: a scientific statement from the American Heart Association. Circulation 2012, 126(11):1408–1428. 10.1161/CIR.0b013e31826890b0 22893607

[5] Casella G, Cassin M, Chiarella F, Chinaglia A, Conte MR, Fradella G, et al: Epidemiology and patterns of care of patients admitted to Italian Intensive Cardiac Care units: the BLITZ-3 registry. J Cardiovasc Med (Hagerstown) 2010, 11(6):450–461. 19952775 10.2459/JCM.0b013e328335233e

[6] Holland EM, Moss TJ: Acute Noncardiovascular Illness in the Cardiac Intensive Care Unit. J Am Coll Cardiol 2017, 69(16):1999–2007. 10.1016/j.jacc.2017.02.033 28427574

[7] Goldfarb M, van Diepen S, Liszkowski M, Jentzer JC, Pedraza I, Cercek B: Noncardiovascular Disease and Critical Care Delivery in a Contemporary Cardiac and Medical Intensive Care Unit. J Intensive Care Med 2017:885066617741873. 10.1177/0885066617741873 29187011

[8] Granger CB, Goldberg RJ, Dabbous O, Pieper KS, Eagle KA, Cannon CP, et al: Predictors of hospital mortality in the global registry of acute coronary events. Arch Intern Med 2003, 163(19):2345–2353. 10.1001/archinte.163.19.2345 14581255

[9] Fonarow GC: Clinical risk prediction tools in patients hospitalized with heart failure. Rev Cardiovasc Med 2012, 13(1):e14–23. 22565534 10.3909/ricm0595

[10] Keegan MT, Gajic O, Afessa B: Severity of illness scoring systems in the intensive care unit. Crit Care Med 2011, 39(1):163–169. 10.1097/CCM.0b013e3181f96f81 20838329

[11] Vincent JL, Moreno R: Clinical review: scoring systems in the critically ill. Crit Care 2010, 14(2):207 10.1186/cc8204 20392287 PMC2887099

[12] Vincent JL, Moreno R, Takala J, Willatts S, De Mendonca A, Bruining H, et al: The SOFA (Sepsis-related Organ Failure Assessment) score to describe organ dysfunction/failure. On behalf of the Working Group on Sepsis-Related Problems of the European Society of Intensive Care Medicine. Intensive Care Med 1996, 22(7):707–710. 8844239 10.1007/BF01709751

[13] Minne L, Abu-Hanna A, de Jonge E: Evaluation of SOFA-based models for predicting mortality in the ICU: A systematic review. Crit Care 2008, 12(6):R161 10.1186/cc7160 19091120 PMC2646326

[14] Argyriou G, Vrettou CS, Filippatos G, Sainis G, Nanas S, Routsi C: Comparative evaluation of Acute Physiology and Chronic Health Evaluation II and Sequential Organ Failure Assessment scoring systems in patients admitted to the cardiac intensive care unit. J Crit Care 2015, 30(4):752–757. 10.1016/j.jcrc.2015.04.014 25981445

[15] Jentzer JC, Bennett C, Wiley BM, Murphree DH, Keegan MT, Gajic O, et al: Predictive Value of the Sequential Organ Failure Assessment Score for Mortality in a Contemporary Cardiac Intensive Care Unit Population. J Am Heart Assoc 2018, 7(6). 10.1161/JAHA.117.008169 PMC5907568 29525785

[16] Afessa B, Keegan MT, Gajic O, Hubmayr RD, Peters SG: The influence of missing components of the Acute Physiology Score of APACHE III on the measurement of ICU performance. Intensive care medicine 2005, 31(11):1537–1543. 10.1007/s00134-005-2751-9 16205890

[17] Herasevich V, Pickering BW, Dong Y, Peters SG, Gajic O: Informatics infrastructure for syndrome surveillance, decision support, reporting, and modeling of critical illness. Mayo Clin Proc 2010, 85(3):247–254. 10.4065/mcp.2009.0479 20194152 PMC2843116

[18] Chandra S, Kashyap R, Trillo-Alvarez CA, Tsapenko M, Yilmaz M, Hanson AC, et al: Mapping physicians' admission diagnoses to structured concepts towards fully automatic calculation of acute physiology and chronic health evaluation score. BMJ Open 2011, 1(2):e000216 10.1136/bmjopen-2011-000216 22102639 PMC3221296

[19] Keegan MT, Gajic O, Afessa B: Comparison of APACHE III, APACHE IV, SAPS 3, and MPM0III and influence of resuscitation status on model performance. Chest 2012, 142(4):851–858. 10.1378/chest.11-2164 22499827 PMC3465106

[20] Aakre C, Franco PM, Ferreyra M, Kitson J, Li M, Herasevich V: Prospective validation of a near real-time EHR-integrated automated SOFA score calculator. Int J Med Inform 2017, 103:1–6. 10.1016/j.ijmedinf.2017.04.001 28550994

[21] Johnson AE, Kramer AA, Clifford GD: A new severity of illness scale using a subset of Acute Physiology And Chronic Health Evaluation data elements shows comparable predictive accuracy. Crit Care Med 2013, 41(7):1711–1718. 10.1097/CCM.0b013e31828a24fe 23660729

[22] Singh B, Singh A, Ahmed A, Wilson GA, Pickering BW, Herasevich V, et al: Derivation and validation of automated electronic search strategies to extract Charlson comorbidities from electronic medical records. Mayo Clin Proc 2012, 87(9):817–824. 10.1016/j.mayocp.2012.04.015 22958988 PMC3538495

[23] Harrison AM, Thongprayoon C, Kashyap R, Chute CG, Gajic O, Pickering BW, et al: Developing the surveillance algorithm for detection of failure to recognize and treat severe sepsis. Mayo Clin Proc 2015, 90(2):166–175. 10.1016/j.mayocp.2014.11.014 25576199 PMC6571011

[24] Rocca WA, Yawn BP, St Sauver JL, Grossardt BR, Melton LJ 3rd: History of the Rochester Epidemiology Project: half a century of medical records linkage in a US population. Mayo Clin Proc 2012, 87(12):1202–1213. 10.1016/j.mayocp.2012.08.012 23199802 PMC3541925

[25] Knox DB, Lanspa MJ, Pratt CM, Kuttler KG, Jones JP, Brown SM: Glasgow Coma Scale score dominates the association between admission Sequential Organ Failure Assessment score and 30-day mortality in a mixed intensive care unit population. J Crit Care 2014, 29(5):780–785. 10.1016/j.jcrc.2014.05.009 25012961 PMC4140959

[26] Toma T, Abu-Hanna A, Bosman RJ: Discovery and integration of univariate patterns from daily individual organ-failure scores for intensive care mortality prediction. Artif Intell Med 2008, 43(1):47–60. 10.1016/j.artmed.2008.01.002 18394871

