# Supplementary figures and images for: Predictive value of individual Sequential Organ Failure Assessment sub-scores for mortality in the cardiac intensive care unit
Source: PLoS One. 2019 May 20;14(5):e0216177. doi: 10.1371/journal.pone.0216177 (PMC6527229; doi:10.1371/journal.pone.0216177)

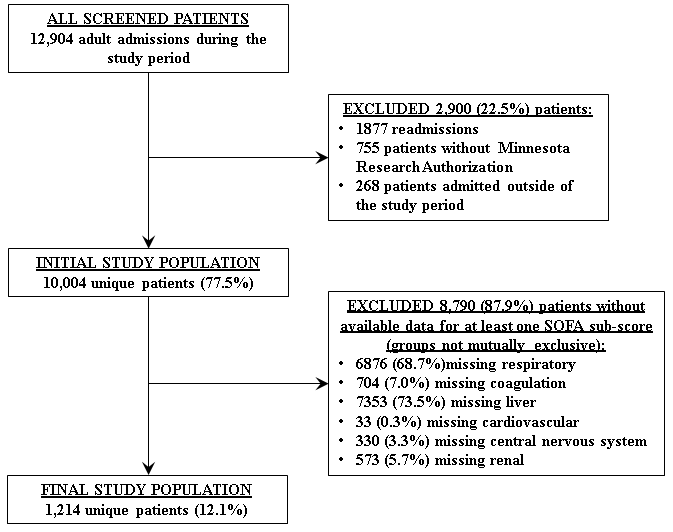

Supplement: S1 Fig — (TIF) [file pone.0216177.s001.tif]

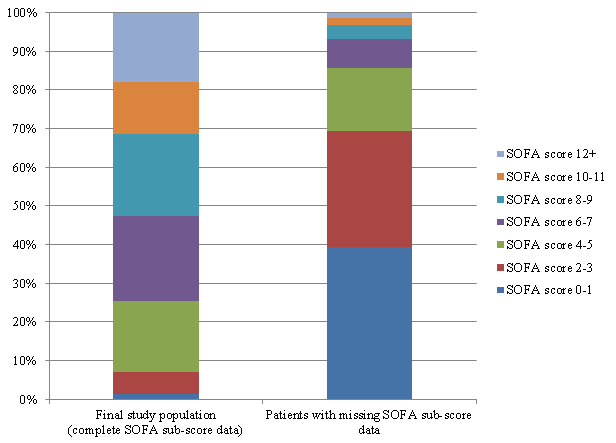

Supplement: S2 Fig — P<0.001 between groups. (TIF) [file pone.0216177.s002.tif]

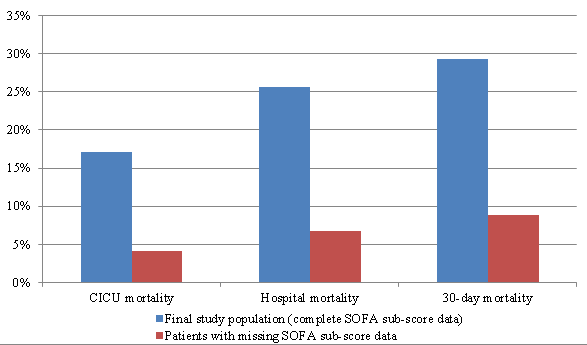

Supplement: S3 Fig — P <0.001 between groups. (TIF) [file pone.0216177.s003.tif]

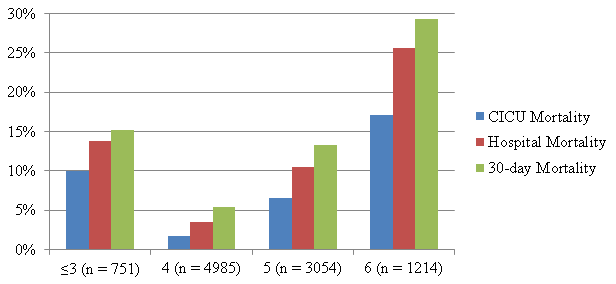

Supplement: S4 Fig — P<0.001 between groups. (TIF) [file pone.0216177.s004.tif]
