# Supplementary material for: Predictive value of individual Sequential Organ Failure Assessment sub-scores for mortality in the cardiac intensive care unit
Source: PLoS One. 2019 May 20;14(5):e0216177. doi: 10.1371/journal.pone.0216177 (PMC6527229; doi:10.1371/journal.pone.0216177)
Supplement: S1 Table — For this analysis, all missing data were imputed as normal. All p values <0.001. (DOCX) [file pone.0216177.s005.docx]

**Supplemental Table 1:** Univariate analysis of Day 1 illness severity scores as predictors of hospital mortality in patients excluded from the final study population due to missing SOFA sub-score data (n = 8790). For this analysis, all missing data were imputed as normal. All p values <0.001.

| **Variable** | **Unit OR** | **95% CI** | **AUROC** |
| --- | --- | --- | --- |
| **APACHE-III** | 1.044 | 1.041-1.048 | 0.77319 |
| **OASIS score** | 1.107 | 1.098-1.116 | 0.76785 |
| **SOFA score** | 1.441 | 1.404-1.480 | 0.80203 |
| **Respiratory SOFA sub-score** | 1.883 | 1.780-1.992 | 0.69802 |
| **Coagulation SOFA sub-score** | 1.308 | 1.156-1.480 | 0.52682 |
| **Liver SOFA sub-score** | 1.504 | 1.235-1.832 | 0.51099 |
| **Cardiovascular SOFA sub-score** | 2.503 | 2.321-2.700 | 0.67664 |
| **CNS SOFA sub-score** | 2.013 | 1.882-2.152 | 0.63958 |
| **Renal SOFA sub-score** | 1.628 | 1.532-1.731 | 0.65366 |
| **SOFA without Respiratory sub-score** | 1.562 | 1.509-1.616 | 0.76912 |
| **SOFA without Coagulation sub-score** | 1.470 | 1.430-1.511 | 0.80571 |
| **SOFA without Liver sub-score** | 1.439 | 1.402-1.477 | 0.80175 |
| **SOFA without Cardiovascular sub-score** | 1.491 | 1.446-1.538 | 0.77873 |
| **SOFA without CNS sub-score** | 1.526 | 1.478-1.574 | 0.78131 |
| **SOFA without Renal sub-score** | 1.490 | 1.447-1.534 | 0.78409 |
| **Simplified SOFA score (3 sub-scores)*** | 1.656 | 1.595-1.721 | 0.77525 |
| **Simplified SOFA score (4 sub-scores)**** | 1.464 | 1.424-1.504 | 0.80293 |

* Cardiovascular, central nervous system, renal

** Cardiovascular, central nervous system, renal, respiratory

Abbreviations: APACHE, Acute Physiology and Chronic Health Evaluation; AUROC, area under the receiver-operator characteristic curve; CNS, central nervous system; OASIS, Oxford Acute Severity of Illness Score; SOFA, Sequential Organ Failure Assessment.
